# Influence of Enzymatically Hydrolyzed Whole Blood Formulated Diet on Nutrient Digestibility, Fecal Bacterial Count, and Immune Responses of Weaned Piglets Challenged with *Escherichia coli* Lipopolysaccharides

**DOI:** 10.3390/life16071049

**Published:** 2026-06-24

**Authors:** Kye Jin Lee, Vetriselvi Sampath, Whajung Cho, In Ho Kim

**Affiliations:** 1 Department of Animal Biotechnology, Dankook University, Cheonan 31116, Republic of Korea; jookyjin1234@naver.com (K.J.L.); vetrio9@dankook.ac.kr (V.S.); 2 Smart Animal Bio Institute, Dankook University, Cheonan 31116, Republic of Korea; 3 Aaminolab, Sejong-si 30006, Republic of Korea; wjcho@aminolab.co.kr

**Keywords:** enzymatically hydrolyzed whole blood protein, immune response, blood plasma replacement, LPS challenge

## Abstract

Weaning stress and immune challenges can negatively affect the health and performance of young pigs by inducing inflammatory responses. Functional protein sources, such as enzymatically hydrolyzed whole blood (EHB), may help alleviate inflammation and improve immune status during stressful conditions. A total of 20 late-weaned pigs [Landrace × Yorkshire × Duroc], 42 days of age, with an initial body weight of 15.34 ± 1.17 kg, were used in a 2-week experiment. Pigs were allotted to a 2 × 2 factorial arrangement with two dietary protein sources [plasma protein (PP) and/or EHB] and two immune challenges (saline or LPS). The four experimental groups were as follows: (1) Plasma-Sal, PP diet + saline injection; (2) Plasma-LPS, PP diet + LPS injection (100 μg/kg BW); (3) EHB-Sal, EHB diet + saline injection; and (4) EHB-LPS, EHB diet + LPS injection (100 μg/kg BW). Each treatment consisted of five replicate pens with one pig/pen. Pigs fed either protein diet with and without LPS showed no (*p* > 0.05) difference in their nutrient digestibility and microbial population. However, pigs challenged with LPS exhibited a higher (*p* < 0.05) rectal temperature, with significant differences observed at 6 h and 12 h post-injection (*p* < 0.001). Dietary effects (*p* < 0.05) were observed for IL-6 and TNF-α concentrations, with pigs fed EHB exhibiting lower values compared with those fed the PP diet following LPS challenge. Consistent with an inflammatory response, pigs challenged with LPS showed elevated (*p* < 0.05) IL-6 and TNF-α concentrations, together with increased white blood cell and lymphocyte counts, at 12 h post-challenge. Moreover, significant (*p* < 0.05) diet × LPS interactions were detected for IL-6 and TNF-α concentrations at 6 h post-challenge, indicating that dietary EHB attenuated the inflammatory response induced by LPS. In summary, a diet formulated with EHB showed a reduced effect of LPS challenge in pigs, making it promising as a functional dietary protein source for improving immune resilience in weaned piglets.

## 1. Introduction

The major protein ingredients used in monogastric animal diets include soybean meal (SBM), fish meal, animal protein products, rapeseed meal, and canola meal [1,2]. However, increasing competition for conventional protein sources, fluctuations in ingredient prices, and concerns regarding the environmental sustainability of feed production have intensified the search for alternative, high-quality protein ingredients for livestock diets [3]. Concurrently, alternative protein sources should possess high nutritional value, excellent digestibility, and the ability to support animal health and performance efficiently [4].

Among alternative protein ingredients, blood-derived products have received considerable attention because of their high crude protein content, balanced amino acid profile, and high nutrient bioavailability [5]. Blood plasma protein (BPP), in particular, contains a wide range of biologically active compounds, including immunoglobulins, albumin, growth factors, peptides, and other functional proteins that can positively influence intestinal health and immune function [6]. Numerous studies have demonstrated that dietary spray-dried plasma improves growth performance, intestinal integrity, and immune status in weaning pigs, especially during periods of physiological and environmental stress [7,8].

Weaning is one of the most critical stages in pig production and is often associated with reduced feed intake, impaired intestinal function, increased susceptibility to enteric pathogens, and activation of inflammatory responses [9]. Although these challenges are most severe immediately after weaning, pigs in the late nursery phase may remain vulnerable to immune and intestinal disturbances, especially under pathogenic or inflammatory stress conditions [10]. Therefore, nutritional strategies that support gut health and immune competence remain important beyond the immediate post-weaning period.

Enzymatic hydrolysis has emerged as a promising processing technology to enhance the functional properties of protein ingredients [11]. During hydrolysis, large protein molecules are broken down into smaller peptides and free amino acids, which can improve protein digestibility and absorption [12]. Furthermore, bioactive peptides generated during enzymatic hydrolysis have been reported to exhibit antimicrobial, antioxidant, anti-inflammatory, and immunomodulatory activities [13]. Previously, Hoque et al. [14] noted that dietary enzymatically hydrolyzed blood (EHB) improved growth performance and nutrient utilization in weaning pigs under normal rearing conditions. Despite the promising findings on the nutritional effects of EHB in newly weaned pigs, its potential role in supporting pigs during pathogenic challenges in the late nursery phase remains unknown. Therefore, we aimed to investigate the effects of dietary plasma protein [PP] and EHB supplementation on immune responses in weaned pigs subjected to an LPS challenge.

## 2. Materials and Methods

The experimental protocol, which conformed to the guidelines of animal welfare (No: DK-1-2330-1), was approved by the Animal Care and Use Committee of Dankook University (Cheonan, Republic of Korea) prior to the trial.

### 2.1. Preparation and Source of EHB and E. coli Lipopolysaccharides

The coagulated porcine blood was collected from a slaughterhouse and crushed using a liquefier. The blood was then treated with 0.3% serine protease. Then it was hydrolyzed at 55 °C for 3 h. The hydrolysate was filtered 3 to 5 times to obtain a clear yellow liquid. Then the filtrate was sterilized at 85 °C for 30 min and powdered using a spray dryer (AminoLab Co. Ltd., Sejong-si, Republic of Korea). For. The *E. coli* LPS (*E. coli*, 0111, B4) was obtained from Sigma-Aldrich (St. Louis, MO, USA).

### 2.2. Experimental Design, Animals, and Diets

A total of 20 late-weaned pigs [Landrace × Yorkshire × Duroc], 42 days of age, with an initial body weight of 15.34 ± 1.17 kg, were used in a 2-week experiment. Pigs were allotted to a 2 × 2 factorial arrangement with two dietary protein sources [PP and/or EHB] and two immune challenges (saline or LPS). The four experimental groups were as follows: (1) Plasma-Sal, PP diet + saline injection; (2) Plasma-LPS, PP diet + LPS injection (100 μg/kg BW); (3) EHB-Sal, EHB diet + saline injection; and (4) EHB-LPS, EHB diet + LPS injection (100 μg/kg BW). Each treatment consisted of five replicate pens with one pig/pen. Experimental diets were formulated to meet or exceed the nutrient requirements recommended by the NRC [15] (Table 1). To create a concentration of 1 mg/mL, the bacterial LPS stock solution was made by dissolving LPS in 0.9% normal saline and stored at −20 °C until use. *n* = 10 pigs received an intraperitoneal injection of LPS at 100 μg/kg BW, whereas the remaining ten pigs received an equivalent volume of sterile saline (0.9% NaCl). All pigs were housed in slatted plastic floor pens individually, identified with ear tags. The room temperature was maintained at 24 °C. All pigs had unlimited access to feed and water through a self-feeder and nipple drinker throughout the experimental period.

### 2.3. Sampling

Individual pig body weight (BW), average daily gain (ADG/pen), average daily feed intake (ADFI/pen), and feed conversion ratio (FCR) were measured at initial, week 2, and overall experimental period. To estimate the apparent total tract nutrient digestibility (ATTD), 0.20% of chromium oxide (Cr_2_O_3_) was mixed with the diets for about a week. At the end of the trial, fresh fecal samples were collected directly from the rectum of each pig by rectal palpation and stored at −20 °C until analysis. Additional fecal samples were collected into microcentrifuge tubes and stored for subsequent analyses. Fasting blood samples (10 mL/pig) were obtained via jugular venipuncture at 0, 6, and 12 h post-injection and collected into vacuum tubes without additives and K3EDTA tubes (Becton Dickinson Vacutainer Systems, Franklin Lakes, NJ, USA). At each sampling time point, rectal temperature was measured and recorded for all pigs.

### 2.4. Laboratory Analysis

Before performing chemical analysis, feed and fecal samples were de-moisturized at 70 °C for 72 h using a J-IDO1 Forced convection drying oven (Seoul, Republic of Korea). Then the feed and fecal samples were ground and sieved through a 1 mm screen to get uniform samples. All samples were analyzed for dry matter (DM), nitrogen (N), and energy (E) following the AOAC protocol [16]. The chromium concentration in feed and fecal samples was measured using a UV spectrophotometer (Optizen POP, Mecasys Co., Ltd., Yuseong-gu, Daejeon, Republic of Korea). The gross energy was measured by an oxygen bomb calorimeter (Parr 6200, Parr Instrument Company, IL, USA). N was determined using a Kjeltec 2300 Nitrogen Analyzer (Foss Analytical, Hillerød, Denmark) following the product manual. The ATTD was estimated using the following formula

ATTD of nutrients (%) = {1 − [(Nf × Cd)/(Nd × Cf)]} × 100,

where Nf is the nutrient concentration in feces (% DM), Nd is the nutrient concentration in diet (% DM), Cd is the chromium concentration in diets, and Cf is the chromium concentration in feces.

### 2.5. Blood Analysis

Peripheral blood mononuclear cells (PBMCs) were isolated from whole blood using density gradient centrifugation. White blood cell (WBC) counts were determined from whole blood samples, and lymphocyte counts were measured from isolated PBMCs using an automated hematology analyzer (ADVIA 120, Bayer, Tarrytown, NY, USA). The proportion of lymphocytes within the total PBMC population was subsequently calculated. Serum samples were obtained by centrifugation at 3000× *g* for 15 min at 4 °C and stored at −80 °C until analysis. Serum concentrations of interleukin-6 (IL-6) and tumor necrosis factor-α (TNF-α) were determined using commercially available porcine ELISA kits (R&D Systems, Minneapolis, MN, USA) according to the manufacturer’s instructions. For flow cytometric analysis, isolated PBMCs were washed with phosphate-buffered saline (PBS) containing 0.5% bovine serum albumin (BSA) and stained with 5 μL of anti-porcine CD4+ and CD8+ monoclonal antibodies (Southern Biotech, Birmingham, AL, USA). The stained cells were analyzed using a FACSCanto II flow cytometer (BD Biosciences, San Jose, CA, USA) to determine the proportions of CD4+ and CD8+ T lymphocytes.

### 2.6. Bacterial Culture

Approximately 1 g of fresh fecal sample was diluted in 9 mL of 1% peptone broth (Becton, Dickinson and Co., Franklin Lakes, NJ, USA) and homogenized thoroughly. Then, 10-fold serial dilutions were prepared and cultured on MacConkey agar plates (Difco Laboratories, Detroit, MI, USA) and *Lactobacillus* medium III agar plates (Medium 638, DSMZ, Braunschweig, Germany). MacConkey agar plates were incubated aerobically at 37 °C for 24 h, while *Lactobacillus* agar plates were incubated anaerobically at 39 °C for 48 h. For each sample, bacterial colonies were counted on plates containing between 30 and 300 colonies. Microbial counts were expressed as log^10^ colony-forming units (CFU)/g of fresh feces.

### 2.7. Statistical Analysis

Data were arranged in a 2 × 2 factorial design and analyzed using two-way ANOVA with GLM procedure of SAS (9.4; SAS institute, USA). The statistical model included the main effects of dietary protein, LPS, and their interaction. The rectal temperature and blood profile variables were measured repeatedly (0, 6, and 12 h post-injection) using the MIXED procedure with time as the repeated factor. Individual pigs were served as an experimental unit. When significant differences were detected, treatment means were separated using Tukey’s HSD test. Statistical significance was declared at *p* < 0.05, whereas 0.05 ≤ *p* < 0.10 was considered a tendency.

## 3. Results

Throughout the experimental period, no significant differences in growth performance were observed among weaned piglets fed diets containing different protein sources and challenged with or without LPS, and thus, data were not included. The effect of different protein diets and LPS challenges on nutrient digestibility of weaned piglets is presented in Table 2. Pigs fed either protein diet or challenged with LPS showed no (*p* > 0.05) difference in their nutrient digestibility of DM, N, and E. Also, there were no (*p* > 0.05) notable changes found in their microbial population (Table 3).

The effect of different protein diets and LPS challenges on rectal temperature of weaned pigs is shown in Table 4. Rectal temperature was not affected by dietary treatment at the beginning of the experiment, and no effect of LPS challenge or diet × LPS interaction was observed at baseline (*p* > 0.05). Whereas 6 h post-injection, pigs challenged with LPS exhibited a higher (*p* < 0.05) rectal temperature than saline-treated pigs (*p* < 0.001), regardless of dietary treatment. Similarly, at 12 h post-injection, LPS-challenged group pigs showed increased (*p* < 0.05) rectal temperature compared with saline-treated pigs.

The effect of different protein diets and LPS challenges on the blood profile of weaned pigs is shown in Table 5. At 6 h post-challenge, LPS administration increased (*p* < 0.05) serum IL-6 and TNF-α concentrations, as well as WBC count, lymphocyte count, and CD4+ and CD8+ T-cell populations in both LPS-challenged groups. Dietary effects (*p* < 0.05) were observed for IL-6 and TNF-α concentrations, with pigs fed EHB exhibiting lower values compared with those fed the PP diet following LPS challenge. Similarly, pigs challenged with LPS showed increased (*p* < 0.05) IL-6, TNF-α, WBC count, and lymphocyte count at 12 h post-challenge. Moreover, significant (*p* < 0.05) diet × LPS interactions were detected for IL-6 and TNF-α concentrations at 6 h post-challenge, indicating that dietary EHB attenuated the inflammatory response induced by LPS.

## 4. Discussion

The weaning period is characterized by physiological and environmental stressors that could increase the susceptibility of pigs to enteric and systemic inflammatory challenges [17]. LPS derived from *E. Coli* is widely used as a model to induce acute inflammation [18] because it activates innate immune pathways through Toll-like receptor 4 signaling, leading to the release of pro-inflammatory mediators and the development of a transient systemic inflammatory response [19]. Thus, the present study confirmed this model as suitable for evaluating nutritional strategies aimed at modulating immune responses in weaned piglets. TNF- α and IL-6 are pro-inflammatory cytokines produced by macrophages and neutrophils, serving as systemic indicators of inflammation in animals [20,21]. An elevated total WBC count indicates the severity of bacterial infection and the occurrence of systemic inflammation [22]. CD4+ and CD8+ are subsets of T lymphocytes, with CD4+ T cells enhancing the adaptive immune system by activating cytotoxic T cells, macrophages, and other immune cells, while CD8+ cells identify pathogens and destroy infected cells [23,24]. The inflammatory and immune responses observed were due to increased production of pro-inflammatory cytokines [25], consistent with previous studies reporting fever and increased inflammation following *E. coli* LPS challenge [26,27]. LPS challenge did not affect the nutrient digestibility of pigs, aligning with findings from a previous study [28]. The bacterial counts of *Lactobacillus* and *E. coli* were unaffected by the LPS challenge. In contrast, a live E. coli challenge typically causes diarrhea and increases the *E. coli* count in pigs [29]. However, since the challenge involved a low-dose, single injection of LPS rather than live bacteria, it only temporarily mimicked the response of a live *E. coli* infection, resulting in no changes in the bacterial count of pigs. One of the notable findings of this study was the attenuation of pro-inflammatory cytokine responses in pigs fed EHB. The mechanism underlying this response remains unclear. However, we suppose that blood-derived protein products contain numerous bioactive components, including immunoglobulins, albumin, peptides, growth factors, and other functional proteins that may influence immune regulation [30]. Previous studies have suggested that plasma protein supplementation modulates inflammatory responses through enhancement of anti-inflammatory pathways, potentially involving cytokines such as IL-10 [31,32]. Although anti-inflammatory markers were not measured in the present study, it was not possible to determine whether a similar mechanism could contribute to the observed reduction in inflammatory cytokines. Interestingly, the EHB diet showed lower expressions of IL-6 and TNF-α compared to the PP diet at both the 6th and 12th h of the LPS challenge. While blood plasma protein specifically contains plasma albumin and globulin, whole blood includes proteins from blood cells and serum. Whole blood fractions comprise plasma protein, serum protein, and blood cells (fibrin and hemoglobin) [33]. The enzymatic digestion, component, and drying process of EHB potentially enhance its nutrient composition, organic iron availability, and amino acid bioavailability compared to blood PP [34,35]. These differences might enable an enzymatically hydrolyzed whole blood diet to combat the LPS challenge to a greater extent. A limitation of the present study is that only selected inflammatory indicators were measured over a relatively short post-challenge period. Additional analyses of anti-inflammatory cytokines, intestinal barrier function, oxidative stress biomarkers, and immune-cell signaling pathways would provide greater insight into the mechanisms responsible for the observed responses. Therefore, further research is needed to clarify how EHB influences immune regulation and whether its beneficial effects extend to conditions involving live pathogenic infections and long-term production performance.

In conclusion, EHB shows promise as a functional dietary protein source for improving immune resilience in weaning pigs. Yet their full biological mechanisms that are responsible for their immunomodulatory effects require further investigation.

## Figures and Tables

**Table 1:** Composition of weaning pig diets.

Item	PP+	EHB+
Corn	41.99	43.82
Soybean meal	16.07	13.88
Fermented Soybean meal	5.00	5.00
Blood plasma protein	6.00	-
Enzymatic hydrolyzed whole blood	-	6.00
Tallow	3.10	3.24
Lactose	14.19	14.19
Whey protein	10.00	10.00
Mono di calcium phosphate	1.11	1.55
Limestone	0.95	0.71
Salt	0.20	0.20
Methionine (99%)	0.23	0.24
Lysine (78%)	0.48	0.49
Mineral mix ^1^	0.20	0.20
Vitamin mix ^2^	0.20	0.20
ZnO (80%)	0.25	0.25
Choline (25%)	0.03	0.03
Total	100.00	100.00
Calculated value		
Crude protein, %	20.00	20.00
ME (kcal/kg)	3450	3450
Ca, %	0.80	0.80
P, %	0.60	0.60
Lys, %	1.60	1.60
Met, %	0.48	0.48
FAT, %	4.88	5.08
Lactose, %	20.00	20.00

^1^ Provided per kg diet: Fe, 100 mg as ferrous sulfate; Cu, 17 mg as copper sulfate; Mn, 17 mg as manganese oxide; Zn, 100 mg as zinc oxide; I, 0.5 mg as potassium iodide; and Se, 0.3 mg as sodium selenite. ^2^ Provided per kilograms of diet: vitamin A, 10,800 IU; vitamin D3, 4000 IU; vitamin E, 40 IU; vitamin K3, 4 mg; vitamin B1, 6 mg; vitamin B2, 12 mg; vitamin B6, 6 mg; vitamin B12, 0.05 mg; biotin, 0.2 mg; folic acid, 2 mg; niacin, 50 mg; D-calcium pantothenate, 25 mg.

**Table 2:** The effect of dietary protein supplementation on nutrient digestibility in weaning pigs challenged with *E. coli* LPS ^1^.

Items, %	Plasma-Sal	Plasma-LPS	EHB-Sal	EHB-LPS	SEM ^2^	*p*-Value		
						Diet Effect	LPS Effect	Interaction
Week 2								
Dry matter	81.01	81.03	81.29	81.22	0.63	0.716	0.972	0.940
Nitrogen	79.35	79.31	79.86	79.83	0.44	0.263	0.932	0.989
Energy	80.05	80.03	80.54	80.59	0.47	0.279	0.976	0.943

^1^ Abbreviation: Plasma-Sal, plasma protein diet + Saline; Plasma-LPS, plasma protein diet + 100 μg/kg LPS; EHB-Sal, enzymatically hydrolyzed whole blood diet + Saline; EHB-LPS, enzymatically hydrolyzed whole blood diet + 100 μg/kg LPS. ^2^ Standard error of means.

**Table 3:** The effect of dietary enzymatically hydrolyzed whole blood supplementation on fecal microbial in weaning pigs challenged with *E. coli* LPS ^1^.

Items, (log^10^)	Plasma-Sal	Plasma-LPS	EHB-Sal	EHB-LPS	SEM ^2^	*p*-Value		
						Diet Effect	LPS Effect	Interaction
Week 2								
*Lactobacillus*	7.01	6.98	7.09	7.11	0.06	0.108	0.885	0.667
*E. coli*	6.45	6.46	6.39	6.40	0.04	0.127	0.878	1.000

^1^ Abbreviation: Plasma-Sal, plasma protein diet + Saline; Plasma-LPS, plasma protein diet + 100 μg/kg LPS; EHB-Sal, enzymatically hydrolyzed whole blood diet + Saline; EHB-LPS, enzymatically hydrolyzed whole blood diet + 100 μg/kg LPS. ^2^ Standard error of means.

**Table 4:** The effect of dietary enzymatically hydrolyzed whole blood supplementation on rectal temperature in weaning pigs challenged with *E. coli* LPS ^1^.

Items °C	Plasma-Sal	Plasma-LPS	EHB-Sal	EHB-LPS	SEM ^2^	*p*-Value ^3^		
						Diet Effect	LPS Effect	Interaction
Rectal temperature								
Initial	39.36	39.30	39.38	39.32	0.14	0.887	0.672	1.000
6 h	39.42 ^b^	40.52 ^a^	39.40 ^b^	40.28 ^a^	0.16	0.428	<0.001	0.501
12 h	39.40 ^b^	40.16 ^a^	39.36 ^b^	39.72 ^ab^	0.23	0.395	0.026	0.310

^1^ Abbreviation: Plasma-Sal, plasma protein diet + Saline; Plasma-LPS, plasma protein diet + 100 μg/kg LPS; EHB-Sal, enzymatically hydrolyzed whole blood diet + Saline; EHB-LPS, enzymatically hydrolyzed whole blood diet + 100 μg/kg LPS. ^2^ Standard error of means. ^3^ Means in the same row with different superscripts differ significantly (*p* < 0.05).

**Table 5:** The effect of dietary enzymatically hydrolyzed whole blood supplementation on blood profile in weaning pigs challenged with *E. coli* LPS ^1^.

Items	Plasma-Sal	Plasma-LPS	EHB-Sal	EHB-LPS	SEM ^2^	*p*-Value ^3^		
						Diet Effect	LPS Effect	Interaction
Initial								
IL-6, pg/mL	91.22	89.46	84.09	85.50	3.89	0.125	0.960	0.649
TNF-α, pg/mL	113.59	116.89	109.98	107.91	4.06	0.204	0.898	0.580
WBC, 10^3^/ μL	17.09	17.11	18.24	18.19	0.91	0.187	0.987	0.969
Lymphocyte, % in total PBMC	57.20	56.94	57.34	57.08	3.51	0.966	0.938	1.000
CD4+, % in total PBMC	13.6	14.6	15.0	14.0	1.2	0.705	1.000	0.350
CD8+, % in total PBMC	19.0	19.2	19.6	18.6	1.1	1.000	0.696	0.559
6 h								
IL-6, pg/mL	107.22 ^c^	801.73 ^a^	110.09 ^c^	742.25 ^b^	11.01	0.018	<0.001	0.010
TNF-α, pg/mL	166.17 ^c^	1045.63 ^a^	171.29 ^c^	973.85 ^b^	14.39	0.038	<0.001	0.019
WBC, 10^3^/ μL	16.96 ^a^	11.96 ^b^	18.08 ^a^	13.60 ^b^	1.08	0.180	0.002	0.796
Lymphocyte, % in total PBMC	57.46 ^b^	71.88 ^a^	57.22 ^b^	72.14 ^a^	2.42	0.997	<0.001	0.927
CD4+, % in total PBMC	14.2 ^c^	21.0 ^b^	14.4 ^c^	25.2 ^a^	1.0	0.102	<0.001	0.134
CD8+, % in total PBMC	18.0 ^c^	39.6 ^b^	18.6 ^c^	43.8 ^a^	1.3	0.075	<0.001	0.172
12 h								
IL-6, pg/mL	109.22 ^b^	432.73 ^a^	95.09 ^b^	398.75 ^a^	11.04	0.024	<0.001	0.319
TNF-α, pg/mL	145.88 ^c^	736.83 ^a^	114.35 ^c^	674.89 ^b^	19.72	0.039	<0.001	0.474
WBC, 10^3^/ μL	17.00 ^ab^	15.16 ^b^	18.02 ^a^	16.28 ^ab^	0.65	0.096	0.009	0.935
Lymphocyte, % in total PBMC	56.96 ^b^	73.32 ^a^	57.22 ^b^	73.42 ^a^	3.28	0.958	0.002	0.981
CD4+, % in total PBMC	14.8	14.6	15.6	15.8	1.1	0.370	1.000	0.855
CD8+, % in total PBMC	18.6	18.4	20.0	20.2	1.1	0.193	1.000	0.867

^1^ Abbreviation: Plasma-Sal, plasma protein diet + Saline; Plasma-LPS, plasma protein diet + 100 μg/kg LPS; EHB-Sal, enzymatically hydrolyzed whole blood diet + Saline; EHB-LPS, enzymatically hydrolyzed whole blood diet + 100 μg/kg LPS. ^2^ Standard error of means. ^3^ Means in the same row with different superscripts differ significantly (*p* < 0.05).

## Data Availability

Data can be available via email to In Ho Kim, inhokim@dankook.ac.kr.
